# Long-term outcomes of sleeve gastrectomy as a revisional procedure after failed gastric band: a multicenter cross-matched cohort study

**DOI:** 10.1007/s13304-021-01182-5

**Published:** 2021-10-03

**Authors:** Francesco de Angelis, Cristian Eugeniu Boru, Angelo Iossa, Nicola Perotta, Fabio Cesare Campanile, Gianfranco Silecchia

**Affiliations:** 1 Division of General Surgery & Bariatric Center of Excellence IFSO-EC, Department of Medico-Surgical Sciences and Biotechnologies, University La Sapienza of Rome, Corso Della Repubblica, 78, 04100 Latina, Italy; 2 Department of General Surgery, “San Pio da Pietralcina” Hospital, Villa D’Agri, Italy; 3 Division of General Surgery, Andosilla Hospital, Civita Castellana, Italy

**Keywords:** Failed gastric banding, Revisional surgery, Sleeve gastrectomy, Weight regain, GERD, Gastric bypass

## Abstract

Laparoscopic adjustable gastric band (LAGB) is the bariatric procedure most likely subject to revisional surgery. Both laparoscopic sleeve gastrectomy (LSG) and Roux-en-Y gastric bypass (LRYGB) represent viable options, but the long-term results are still lacking. In 2014, we published the 2-year follow-up of our multicenter cohort of revisional LSG after failed LAGB. Evaluate the long-term follow-up (median 9.3 years) of the same cohort of patients. University and primary-care hospitals, Italy. We retrospectively examined a prospectively maintained database of the previously published multicenter cohort of 56 patients who underwent LSG after failed LAGB between 2008–2011. The control group included cross-matched non-revisional LSGs. The primary endpoint was weight loss, secondary endpoints co-morbidities, and the need for further bariatric surgery. The study group included 44 patients and the control group 56. We found %EWL 53% Vs. 67% (*p* = .021), %EBMIL (54 Vs. 68%, *p* = .018), %TWL (26 Vs. 34%, *p* = .002). We also found more severe GERD (gastroesophageal reflux disease) symptoms in the revisional than in the primary group (9.0 vs. 1.8% mild and 23.0 vs. 3.0% severe). Ten patients from the revisional group (22.7%) vs. eight in the primary group (13%) underwent further bariatric surgery (LRYGB). Our results showed less favorable weight loss in revisional than primary LSG after LABG, higher prevalence of GERD, and a more frequent need for further revisional surgery. Despite the study’s limitations, the present data suggest that the long-term outcomes may offset the possible reduced short-term complication rate after revisional sleeve gastrectomy for a failed LABG.

## Introduction

Laparoscopic adjustable gastric band (LAGB) is the bariatric procedure most likely subject to revisional surgery [1]. Despite good short-term outcomes in weight loss and postoperative complications, it is associated with over 50% long-term failure [1, 2]. Although revisional surgery is considered safe and effective, there is still considerable heterogeneity in choosing the proper intervention after a failed primary procedure [3, 4]. Both laparoscopic Roux-en-Y gastric bypass (LRYGB) and laparoscopic sleeve gastrectomy (LSG) represent viable options for failed LAGB; however, the long-term results for both revisional procedures are still lacking [5, 6]. To our knowledge, there are no multicenter cohort studies with a long-term follow-up after revisional LSG following LAGB [7].

In 2014, we published the 2-year follow-up of our multicenter cohort of revisional LSG in a “2-step” approach after failed LAGB. In 56 patients, we reported excess weight loss (%EWL) of 78.5% without major complications. The results were not statistically different from those obtained in our primary (not revisional) sleeve gastrectomy series [8].

The present, multicenter, retrospective cohort study aimed to update the long-term results of LAGB patients converted to LSG, providing a 9-year follow-up of that series.

## Methods

We retrospectively examined a prospectively maintained database of the previously published cohort (retrospective cohort study) [8]. Three Italian bariatric centers were involved in the study, which was approved by the Institutional Review Board (SCDSBMC/27.05.2020 University La Sapienza of Rome) and the Local Committees. The same 56 patients of the published short-term study were included in the present study group; they underwent LSG after failed LAGB between 2008 and 2011 (“revisional group”). The results were compared to patients selected from the original study’s not-revisional control group, cross-matched for age, sex, weight, and BMI (“primary group”).

Revisional surgery had been indicated for inadequate EWL (< 30%), weight regain, and LAGB-related complications. The choice for LSG as a revisional procedure was based on the multidisciplinary re-evaluation of the alimentary and lifestyle behavior and absence of gastroesophageal reflux disease (GERD). The preoperative workup included upper gastro-intestinal contrast studies and endoscopy. No hiatal hernia was found at LSG.

**Fig. 1:**
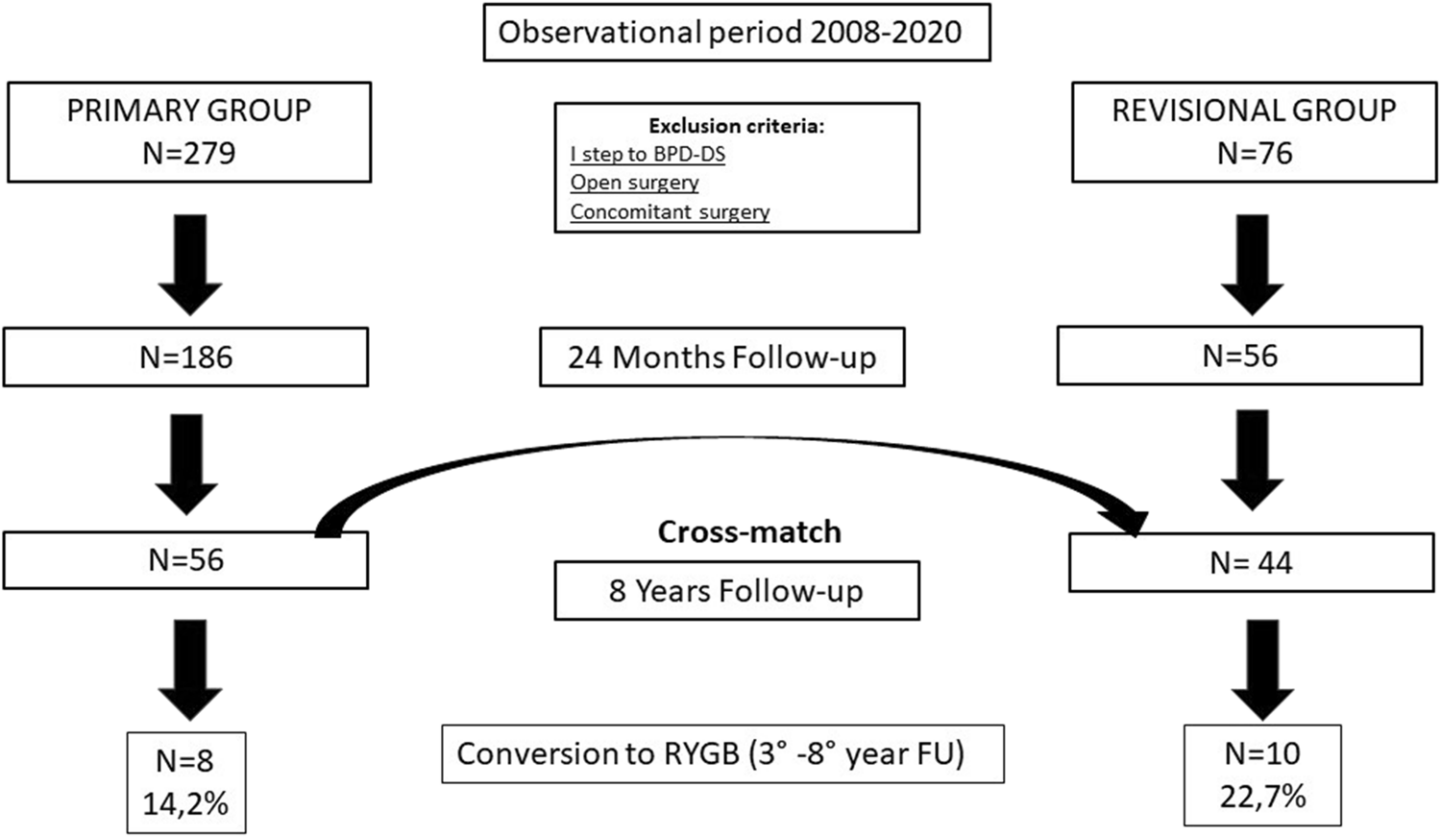
Study’s flow-chart of revisional LSG after failed gastric banding vs. primary cross-matched LSG

The surgical technique for band removal, sleeve gastrectomy, and perioperative management was similar in all centers. The exclusion criteria, demographics, and the study flow-chart are reported in Table 1 and Fig. 1. All patients were evaluated yearly by a multidisciplinary team. The gastroesophageal reflux disease GERD Health-Related Quality of Life Questionnaire (GERD-HRQL) was administered yearly starting from 2016, to all operated patients, during the scheduled outpatient evaluation [9]. GERD symptoms were defined as absent when patients reported a GERD-HRQL score of 0, mild from 1 to 15, moderate from 16 to 30, and severe from 31 to 50.

**Table 1:** Demographics of revisional LSG after failed band vs. cross-matched primary LSG

Characteristics	Revisional group	Primary group	*p* value
Number	44	56	
Gender			
Male	10 (22.7%)	8 (17.7%)	
Female	34 (77.3%)	48 (82.3%)	
Age: median (range)	46 (21–59)	42 (20–59)	*p* = .600
Weight in Kg: median (range)	114 (90–175)	124 (98–213)	*p* = .056
BMI (Kg/m^2^): median	42.51 (32.6–56.5)	44.41 (37–54)	*p* = .161
Comorbidities			
Diabetes Type II	7 (15.9%)	11 (19.64%)	*p* = .632
Hypertension	20 (45.45%)	24 (42.85%)	*p* = .661

Insufficient weight loss was defined as EWL < 50% one year postoperatively; weight regain as an increase of more than 10 kg from nadir (lowest weight obtained) [10].

All patients that underwent further revisional surgery had a multidisciplinary re-evaluation (nutritional and psychological counseling included), upper GI endoscopy, contrast X-ray. Esophageal manometry and pH-metry were obtained in selected cases, or a CT scan with 3D reconstruction to rule out a hiatal defect or intrathoracic sleeve migration [11]. Non-responder GERD patients were referred for conversion to Roux-en-Y Gastric Bypass (RYGB), with posterior cruroplasty indicated. If the hiatal defect was associated with weak pillars, a biosynthetic, absorbable scaffold prosthesis was added to the cruroplasty [12].

The primary endpoint was weight loss after revisional LSG in terms of percent excess weight loss (%EWL), percent of total weight loss (%TWL), percent excess BMI loss (%EBMIL) [13]. Secondary endpoints were resolution, relapse, or de novo occurrence of major co-morbidities and the need for further bariatric procedures after failed revisional surgery for any reason.

The statistical analysis was performed using Statistica10 (TIBCO Software Inc., CA, USA). Normally distributed quantitative variables were expressed as mean with standard deviation, median with the min–max range in skewed data, qualitative variables as a percentage. The Student *t-*test and Chi-Square test were used to compare continuous and categorical data. *p* values < 0.05 (two tailed) were considered significant.

## Results

After a median follow-up of 9.3 years (range 8.0–12.8 years), 44 out of the initial 56 patients included in the first report were available for the study’s completion (10 males and 34 females). Their median initial BMI was 42.51 kg/m^2^ (range 32.6–56.5). No patient died during the follow-up. Twelve patients were unavailable for the study (one for a severe disability, two for cognitive impairment due to senile dementia and nine for refusal to participate in the scheduled controls).

The matched control group included 56 not-revisional patients (8 males and 48 females) with a median BMI of 44.41 kg/m^2^ (range 37–54). All of them were available for follow-up.

The %EWL, %EBMIL and %TWL were significantly different and indicated lower weight loss after revisional surgery. The data are shown in Table 2.

**Table 2:** EWL%, TWL% and EBMI% after 8 years follow-up of revisional LSG after failed band vs. cross-matched primary LSG

	RG 8 year	PG 8 year	*p* value
EWL %	53 ± 26	67 ± 27	0.021
TWL %	26 ± 12	34 ± 12	0.002
EBMIL %	54 ± 25	68 ± 27	0.018

Data are expressed as mean ± SD

*EWL* Excess weight loss, *TWL* Total weight loss, *EBMIL* Excess BMI loss, *RG* Revisional group, *PG* Primary group

The GERD-HRQL questionnaires’ analysis reported that in the revisional group, 30 patients (68%) were asymptomatic, 4 (9%) mildly and 10 (23%) severely symptomatic. In the primary group, there were 53 (95%) asymptomatic, 1 (1.8%) mildly and 2 (3%) severely symptomatic patients (*p* = 0.01). In the revisional group, 34% of patients used proton pump inhibitors (PPIs) to treat GERD (either self-medication or prescribed by the general practitioner), vs. only 8 (12%) of primary group.

Ten patients from the revisional group (22.7%) and eight from the primary group (14%) underwent further laparoscopic RYGB. The indication to re-revision was insufficient weight loss or weight regain (2 vs. 5 patients) and severe GERD associated with a grade B esophagitis or higher (8 vs. 3 patients) [13, 14]. A concomitant type I hiatal hernia was detected in 5 revisional patients and none of the controls.

At the end of the follow-up, hypertension was present in 18.1% of the revisional vs. 5.3% of the primary group (preoperative rate: 45.4 vs. 42.8%), and type II diabetes in 2.3% Vs. nil (preoperative rate: 15.9 vs. 19.6%).

## Discussion

The long-term follow-up (median: 9.3 years) of our series of revisional LSG following LAGB demonstrated lower weight loss than in the primary LSG control group and a higher prevalence of severe GERD. GERD was also the main reason to convert a revisional sleeve gastrectomy to RYGB (8 out of 10 in the revisional group vs. 3 out of 8 in the primary group).

LSG and RYGB have been proposed as effective revisional procedures after failed LAGB. Both show good short-term outcomes and acceptable postoperative complication rates [1–6]. It is controversial which of the two procedures is more effective on long-term (> 5 years) weight loss, co-morbidities control, and complications [3, 6].

Angrisani et al. found no statistically significant difference in percent excess weight loss in patients converted to LSG or RYGB [15]. Other studies showed less weight loss with conversion to LSG when compared with RYGB [16] and less weight loss than primary LSG, as in our present experience.

Recent evidence supports that single-step and two steps approaches are both safe [16, 17]. In both approaches, conversion to LSG might determine fewer short-term post-op complications than RYGB [4, 17].

In 2014, we evaluated the short-term follow-up of a cohort that underwent revisional LSG after failed LAGB in a “2-step” approach; the results demonstrated a good weight loss at 24 months, associated with low major complications rate.

The longer follow-up of the same series, included in the present work, provides evidence that de novo, severe GERD manifests as a mid-term complication and often requires further revisional surgery. Similar findings were shown in non-revisional sleeve gastrectomy [18, 19].

Our findings suggest that when conversion to LSG rather than RYGB is selected, the burden of long-term GERD may balance the advantage of a lower short-term complication rate.

The finding of type I hiatal hernia in 50% of our revisional patients who underwent conversion to RYGB deserves attention. We did not find any hiatal hernia in patients who had RYGB after a failed primary LSG. The significantly higher prevalence in the study group can hardly be related to a lack of hiatus dissection at the previous LSG: even if an occasional hiatal hernia could have been missed, we systematically explored the gastroesophageal junction at the conversion surgery, as previously described. One hypothesis is that the extensive dissection, due to the scarring of the previous gastric band site, could have weakened the hiatal area and compromised some of the natural contention methods.

In the future, the difference between the two groups in terms of “de novo” GERD incidence, despite the systematic hiatus exploration, may influence the revisional operation’s choice. The patients who required LSG’s revisions to RYGB did not show any postoperative complications in both groups, but other series’ rate is up to 25% [19–22].

The present study includes all the known limitations of the small-size retrospective design. The case-matching could also have missed relevant confounding factors. In addition, the high (21.4%) dropout rate from the previous study group could have introduced a selection bias and limited the ability to control confounding control by the matching design. Finally, as per an intention-to-treat design, we administered the GERD-HRQL questionnaire at the end of the study period, regardless of the patient having had an additional revising procedure (RYGB) or not. This aspect affected the results about GERD symptoms.

However, this is the first long-term multicentric retrospective study on revisional 2-stage LSGs after failed LAGB. Moreover the prospectively maintained database available for the entire follow-up, adds strengthens the study’s design.

## Conclusion

Our results confirmed less favorable weight loss in revisional than primary LSG after LABG. In the revisional group, the incidence of GERD and the need for further revisional surgery are higher. Despite the study’s limitations, the present data suggest that the long-term outcomes may offset the possible reduced short-term complication rate after revisional sleeve gastrectomy. Short- and long-term consequences should be included in an exhaustive discussion with the patient candidate to the revision.

## Data Availability

On request.
